# Maltol has anti-cancer effects via modulating PD-L1 signaling pathway in B16F10 cells

**DOI:** 10.3389/fphar.2023.1255586

**Published:** 2023-09-05

**Authors:** Na-Ra Han, Hi-Joon Park, Seong-Gyu Ko, Phil-Dong Moon

**Affiliations:** ^1^ College of Korean Medicine, Kyung Hee University, Seoul, Republic of Korea; ^2^ Korean Medicine-Based Drug Repositioning Cancer Research Center, College of Korean Medicine, Kyung Hee University, Seoul, Republic of Korea; ^3^ Department of Anatomy and Information Sciences, College of Korean Medicine, Kyung Hee University, Seoul, Republic of Korea; ^4^ Department of Preventive Medicine, College of Korean Medicine, Kyung Hee University, Seoul, Republic of Korea; ^5^ Center for Converging Humanities, Kyung Hee University, Seoul, Republic of Korea

**Keywords:** maltol, PD-L1, melanoma, cisplatin, CTLL-2 cells, B16F10 cells

## Abstract

**Introduction:** Among skin cancers, melanoma has a high mortality rate. Recent advances in immunotherapy, particularly through immune checkpoint modulation, have improved the clinical treatment of melanoma. Maltol has various bioactivities, including anti-oxidant and anti-inflammatory properties, but the anti-melanoma property of maltol remains underexplored. The aim of this work is to explore the anti-melanoma potential of maltol through regulating immune checkpoints.

**Methods:** The immune checkpoint PD-L1 was analyzed using qPCR, immunoblots, and immunofluorescence. Melanoma sensitivity towards T cells was investigated via cytotoxicity, cell viability, and IL-2 assays employing CTLL-2 cells.

**Results:** Maltol was found to reduce melanin contents, tyrosinase activity, and expression levels of tyrosinase and tyrosinase-related protein 1. Additionally, maltol suppressed the proliferative capacity of B16F10 and induced cell cycle arrest. Maltol increased apoptotic rates by elevating cleaved caspase-3 and PARP. The co-treatment with maltol and cisplatin revealed a synergistic effect on inhibiting growth and promoting apoptosis. Maltol suppressed IFN-γ-induced PD-L1 and cisplatin-upregulated PD-L1 by attenuating STAT1 phosphorylation, thereby enhancing cisplatin’s cytotoxicity against B16F10. Maltol augmented sensitivity to CTLL-2 cell-regulated melanoma destruction, leading to an increase in IL-2 production.

**Discussion:** These findings demonstrate that maltol restricts melanoma growth through the downregulation of PD-L1 and elicits T cell-mediated anti-cancer responses, overcoming PD-L1-mediated immunotherapy resistance of cisplatin. Therefore, maltol can be considered as an effective therapeutic agent against melanoma.

## Introduction

Melanoma is considered to be one of the deadliest cancers in the world (Guergueb and Akhloufi, 2021). The mortality and incidence of melanoma have increased rapidly over the past few decades, and cases are growing faster than any other type of solid cancer (Ko, 2017). Recent advances in immunotherapy have greatly improved the clinical treatment of melanoma and the blockade of immune checkpoints regulated by programmed death 1 (PD-1) and its corresponding PD ligand 1 (PD-L1) antibodies has effectively reactivated the immune-mediated elimination of melanocytes (Greil et al., 2017).

PD-1, which is a cell surface receptor on T cells, operates as a checkpoint that regulates T cell exhaustion. The binding between PD-1 and PD-L1 serves to suppress T cell activation, leading to immune suppression (Jiang et al., 2019). Interaction between PD-1 and PD-L1 subsequently inhibits the cytotoxicity of cytotoxic T lymphocytes (CTLs, Jiang et al., 2019). Atezolizumab, an anti-PD-L1 antibody, is used in melanoma patients because it is safe and has anti-tumor activity (de Azevedo et al., 2021). Notably, IFN-γ acts as a critical cytokine in tumor microenvironments, and this can contribute to tumor immune evasion by potently inducing PD-L1 expression in tumor cells and impairing CTL-immune responses (Mandai et al., 2016; Jorgovanovic et al., 2020). Unfortunately, the chronic presence of IFN-γ in inflammatory tumor microenvironments fails to contribute to tumor cell eradication and instead induces tumorigenesis (Mojic et al., 2017).

The disadvantages of conventional cancer chemotherapy and the advantages of more natural treatment options are driving the interest in the use of complementary and alternative medicines (Molassiotis et al., 2005). Accumulating evidence has revealed that phytochemical compounds hold potential as anti-cancer drugs (Chinembiri et al., 2014). Several phytochemical compounds have exhibited anti-cancer effects with promising effects on the PD-1/PD-L1 checkpoints (Xu et al., 2018; Ravindran Menon et al., 2021). Maltol, a type of phenolic compound among the phytochemicals, is typically used as a flavor enhancer and often as a marker for the quality control of various ginseng products (Jeong et al., 2015; Jin et al., 2023). Maltol is known to be effective in treating inflammation (Ahn et al., 2022), oxidation (Yang et al., 2006; Mao et al., 2022), aging (Sha et al., 2021), nephrotoxicity (Mi et al., 2018), and liver damage (Liu et al., 2018). Regarding the anti-cancer effects of maltol, anti-cancer effects of maltol-containing complexes or maltol itself in relation to promyelocytic leukemia and oral squamous cell carcinoma cell lines have been reported (Yasumoto et al., 2004; Notaro et al., 2020). However, the anti-cancer properties of maltol concerning melanoma have not been comprehensively studied.

In this work, we investigated the anti-cancer potential of maltol against melanoma cells, as well as its regulatory effects on melanogenesis. Apart from elucidating the growth-inhibiting and pro-apoptotic properties of maltol on melanoma cells by downregulating PD-L1 expression, our observations also showed that maltol was capable of enhancing T cell-regulated eradication of melanoma cells.

## Materials and methods

### Chemicals and reagents

Maltol (purity ≥99%) was obtained from Sigma-Aldrich Co. (St. Louis, MO, United States); cisplatin (purity ≥99.7%), α-melanocyte-stimulating hormone (α-MSH), and L-dopa from MedChemExpress (Monmouth Junction, NJ, United States); recombinant IFN-γ from R & D system Inc., (Minneapolis, MN, United States); antibodies specific for tyrosinase (sc-20035), tyrosinase related protein 1 (TYRP1, sc-166857), caspase-3 (sc-7148), PARP (sc-365315), PD-L1 (sc-518027), phosphorylated signal transducer and activator of transcription 1 (pSTAT1, sc-8394), STAT1 (sc-464), actin (sc-8432), and GAPDH (sc-32233) from Santa Cruz Biotechnology, Inc. (Dallas, Texas, United States); anti-tubulin antibody (3,873) from Cell Signaling Technology (Danvers, MA, United States); anti-mouse IgG H&L (Alexa Fluor^®^ 647, ab150115) from abcam (Cambridge, MA, United States); anti-IL-2 antibodies (554424, 554426) from BD Biosciences (San Jose, CA, United States).

### Cell culture

The B16F10 (mouse) and A375 (human) melanoma cell lines were purchased from the Korean Cell Line Bank (Seoul, Republic of Korea). The CTLL-2 cell line (# TIB-214) was purchased from the American Type Culture Collection (ATCC, Manassas, VA, United States). B16F10 and A375 cells were maintained in Dulbecco’s modified Eagle’s medium (Gibco, Waltham, MA, United States) supplemented with 10% fetal bovine serum (Merck Millipore, Burlington, MA, United States) and 1% penicillin/streptomycin (Gibco). CTLL-2 cells were cultured in RPMI 1,640 medium (ATCC), supplemented with 10% T cell culture supplement containing concanavalin A (T-STIM with Con A, BD Biosciences), 10% fetal bovine serum, and 1% penicillin/streptomycin.

### Melanin content and tyrosinase activity assay

Melanin content and tyrosinase activity assessments were carried out as described previously (Zhou et al., 2021; Bodurlar and Caliskan, 2022). B16F10 cells (1 × 10^5^/well) were exposed to maltol and then treated with α-MSH (100 nM) for 72 h. For the melanin content assay, the cell pellets were dissolved in 1 N NaOH containing 10% dimethyl sulfoxide (DMSO) and subjected to cell lysis for 1 h at 80°C. The optical density of melanin content was spectrophotometrically measured with a microplate reader (405 nm). In the tyrosinase activity assay, the cell pellets were lysed in PBS containing 1% Triton X-100 for 2 h at 80°C. A freshly prepared substrate (L-DOPA, 10 mM) was then added and incubated for 30 min at 37°C. The resulting absorbance was spectrophotometrically analyzed with a microplate reader (475 nm).

### Cell viability assay

Cell proliferation was analyzed by using an MTT [3-(4,5-dimethylthiazol-2-yl)-2,5-diphenyltetrazolium bromide tetrazolium] assay. B16F10 cells and A375 cells were treated with maltol or cisplatin for 48 h. Following this, MTT solution (5 mg/mL) was added to each well, and the resulting formazan crystals were dissolved in DMSO. The absorbance was measured with a microplate reader (570 nm).

### Flow cytometric analysis

For cell cycle analysis, maltol- or cisplatin-treated B16F10 cells were fixed in ice-cold 70% ethanol overnight at 4°C. Then, the cells were incubated in PBS buffer containing RNase (100 μg/mL) and propidium iodide (PI, 50 μg/mL) for 30 min at room temperature, utilizing a PI flow cytometry kit (abcam, Cambridge, United Kingdom). For apoptosis analysis, B16F10 cells treated with maltol or cisplatin were stained with annexin V and PI using a FITC annexin V apoptosis detection kit with PI (Biolegend, San Diego, CA, United States). Cell surface PD-L1 detection was performed with reference to previous protocols (Tang et al., 2018; Xu et al., 2018). Maltol or cisplatin-treated B16F10 cells were first blocked with anti-CD16/32 (anti-FcγIII/II receptor, clone 2.4G2, BD Biosciences, ≤ 1 µg/million cells), then incubated with PE-conjugated anti-mouse PD-L1 antibody (12-5982-83, eBioscience, San Diego, CA, United States, 1:200) in FACS buffer (PBS containing 0.1% BSA and 0.02% NaN_3_) for 30 min at 4°C. Following PBS washes, the samples were resuspended in FACS buffer and analyzed through flow cytometry.

### Immunoblots

Western blotting was carried out as described previously (Han et al., 2022b). The primary antibodies were used at a 1:500 dilution, and the secondary antibodies were used at a 1:10,000 dilution.

### Quantitative PCR analysis

Real-time quantitative PCR (qPCR) was conducted as described previously (Han et al., 2022b). The primer sequences are as follows: mPD-L1 (For: 5′-TGC​TGC​ATA​ATC​AGC​TAC​GG-3′, Rev: 5′-GCT​GGT​CAC​ATT​GAG​AAG​CA-3′) and mGAPDH (For: 5′-CCA​ATG​TGT​CCG​TCG​TGG​ATC​T-3′, Rev: 5′-GTT​GAA​GTC​GCA​GGA​GAC​AAC​C-3′); hPD-L1 (For: 5′-ATT​TGG​AGG​ATG​TGC​CAG​AG-3′, Rev: 5′-CCA​GCA​CAC​TGA​GAA​TCA​ACA-3′) and hGAPDH (For: 5′-TCG​ACA​GTC​AGC​CGC​ATC​TTC​TTT-3′, Rev: 5′-ACC​AAA​TCC​GTT​GAC​TCC​GAC​CTT-3′).

### Immunofluorescence

Immunofluorescence was carried out as described previously (Han et al., 2022a). The antibody specific for PD-L1 was used at a 1:500 dilution, and anti-mouse IgG H&L (Alexa Fluor^®^ 647) was used at a 1:1,000 dilution.

### Co-culture experiments

After B16F10 cells (target cells, 2 × 10^4^/well) were adhered to the plates, CTLL-2 cells (effector cells, 4 × 10^5^/well) were added to each well. Following cell stabilization, maltol was administered to the target cells or co-cultured cells for 24 h. For the viability assay, the remaining viable target cells were exposed to an MTT solution. Cytotoxic activity against the target cells was measured in cell-free supernatants using a cell cytotoxicity assay kit (DoGenBio, Seoul, Republic of Korea). For the IL-2 release assay, the co-cultured cells were subjected to maltol and IFN-γ (10 ng/mL) treatment for 48 h. The IL-2 release from the co-cultured cells was assessed through an enzyme-linked immunosorbent assay (ELISA) according to the manufacturer’s instructions. The IL-2 capture antibody was used at a 1:500 dilution, and IL-2 detection antibody was used at a 1:1,000 dilution.

### Statistical analysis

The error bars in the figures represent the standard error of the mean (SEM). An unpaired Student’s *t*-test was employed to compare two groups, and ANOVA followed by the Türkiye *post hoc* test was used to discern differences among several groups, all facilitated through IBM SPSS software (Armonk, NY, United States). The graphical representation was prepared using Prism—GraphPad (Boston, MA, United States). Statistical significance is denoted as *p < 0.05, **p < 0.01, and ***p < 0.001.

## Results

### Regulatory effect of maltol on melanin content in B16F10 melanoma cells

**FIGURE 1:**
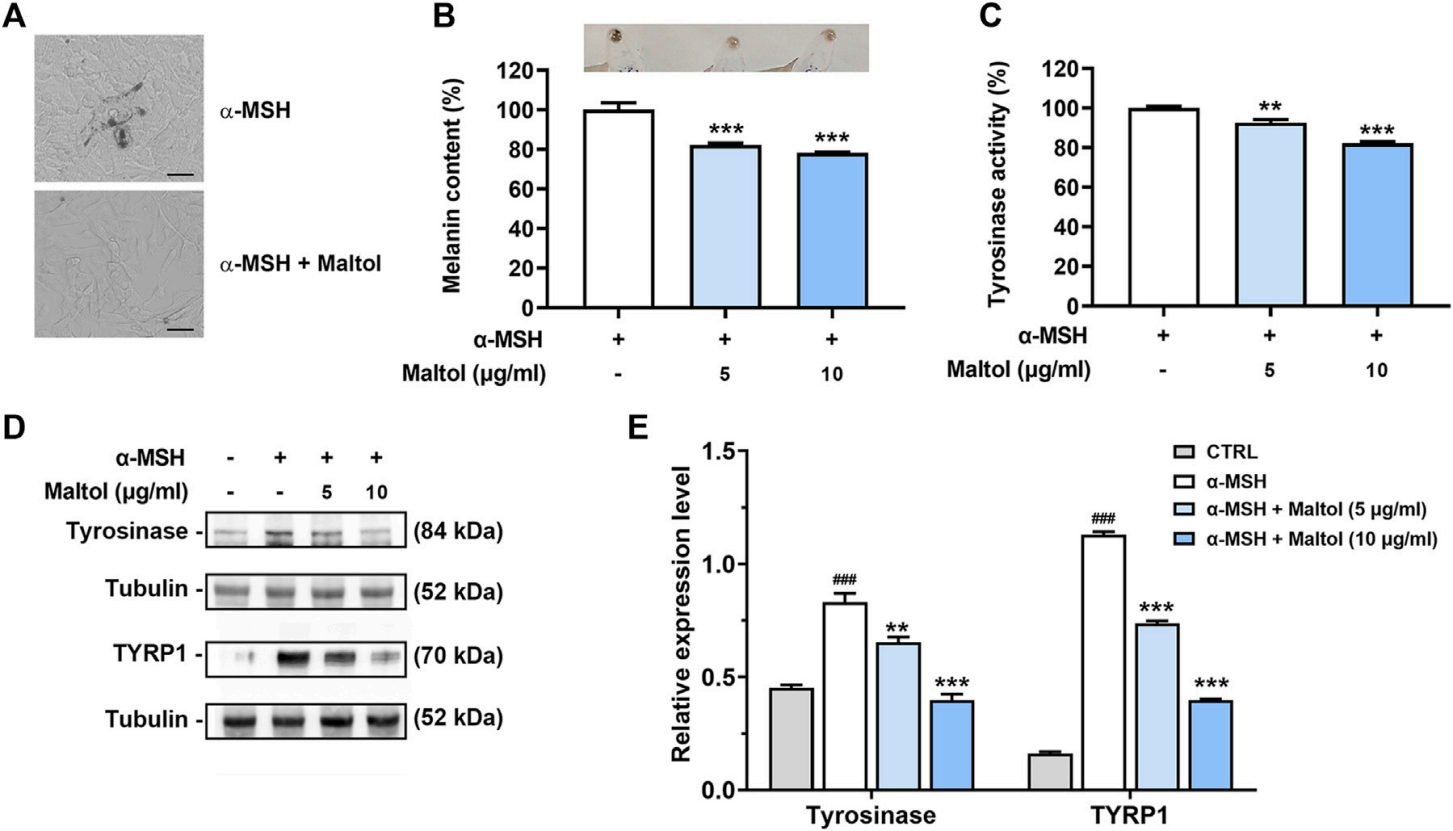
Effects of maltol on melanin content. **(A)** Maltol was pre-treated and α-MSH (100 nM) was then treated for 72 h in B16F10 cells. Representative images of dark pigmentation in α-MSH-stimulated group and α-MSH and maltol (10 μg/mL)-treated group were observed in B16F10 cells (scale bar = 50 μm). **(B)** The relative melanin content in α-MSH-stimulated B16F10 cells was measured as mentioned in the *Materials and methods* section (*n* = 4 per group). Pellets of B16F10 aggregates from each group were imaged above. **(C)** Intracellular tyrosinase activity in α-MSH-stimulated B16F10 cells was analyzed as mentioned in the *Materials and methods* section (*n* = 4 per group). **(D)** Maltol was pre-treated and α-MSH (100 nM) was then treated for 48 h in B16F10 cells. The tyrosinase and TYRP1 levels were assessed by Western blot analysis. Tubulin was a loading control. **(E)** Graphs show quantification of relative band intensities for tyrosinase and TYRP1 (*n* = 3 per group). ^**^ *p* < 0.01 and ***p < 0.001 vs. α-MSH-stimulated group.

Melanogenesis has been linked to melanoma progression (Slominski et al., 2015). We examined whether maltol could affect the melanin content in B16F10 cells. Melanogenesis induced by α-MSH begins with tyrosine oxidation catalyzed by tyrosinase (Chen et al., 2014). As shown in Figures 1A, B, dark pigmentations were observed upon α-MSH treatment, indicating melanin synthesis, while maltol attenuated the dark pigmentations, resulting in a decrease in melanin content (*p <* 0.05). The color of the pellet was also darkened by α-MSH, while it became lighter in the maltol-treated group (Figure 1B). Maltol significantly suppressed the levels of tyrosinase activity increased by α-MSH (*p <* 0.05; Figure 1C). We further investigated whether maltol would inhibit the expression of key regulatory molecules on melanogenesis, such as tyrosinase and TYRP1 (Chen et al., 2014). The expression levels of tyrosinase and TYRP1 were also significantly decreased by maltol (*p* < 0.05; Figures 1D, E).

### Growth-inhibiting effect of maltol in B16F10 melanoma cells

**FIGURE 2:**
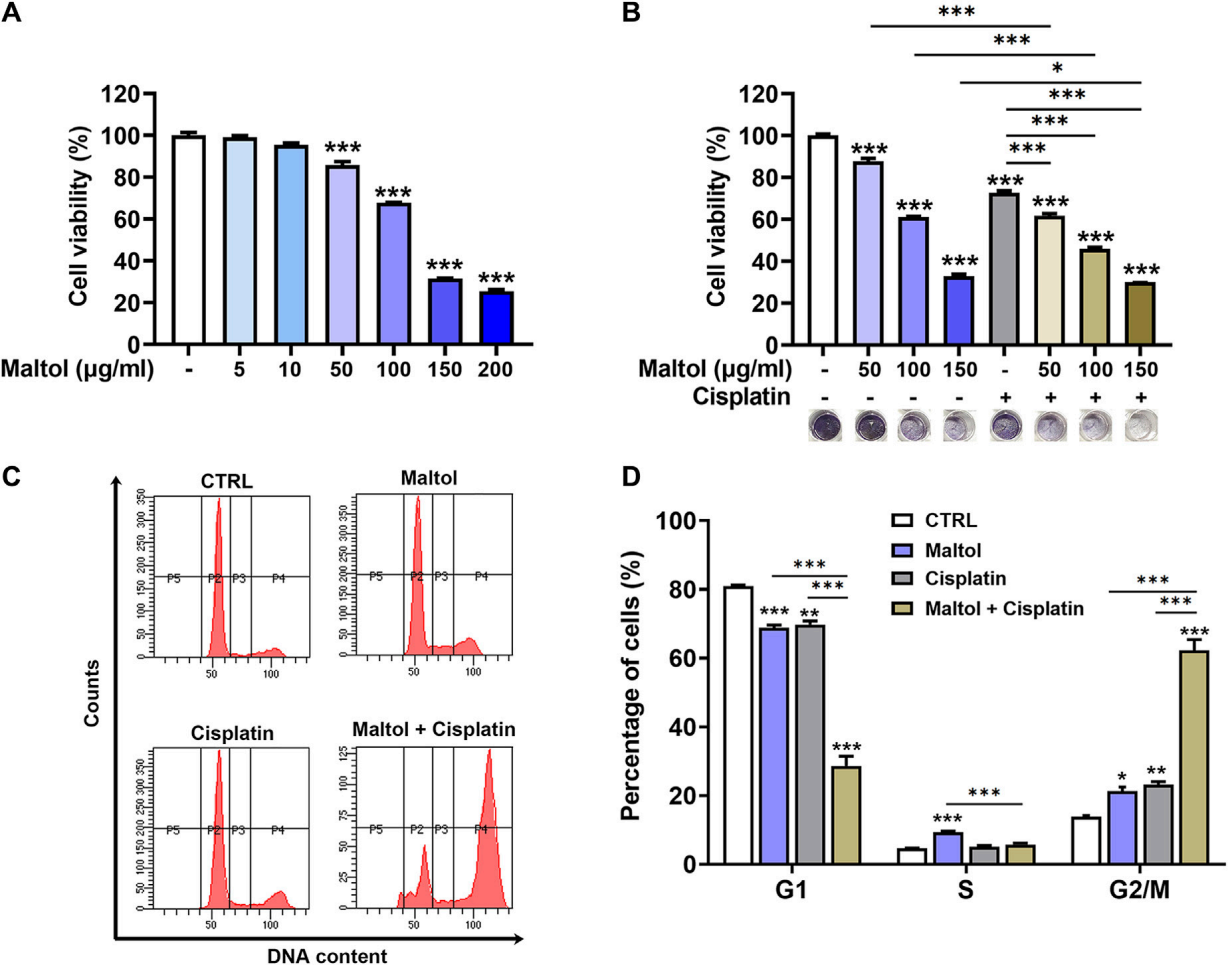
Effects of maltol on growth of B16F10 melanoma cells. **(A)** Maltol was treated in B16F10 cells at various concentrations for 48 h. The cell viability was measured with an MTT assay (*n* = 4 per group). **(B)** Maltol or cisplatin (20 µM) were treated in B16F10 cells for 48 h (*n* = 6 per group). Representative MTT-formazan from each group was imaged below. **(C)** Maltol or cisplatin were treated in B16F10 cells for 48 h. Cell cycle distribution was assessed by flow cytometry. **(D)** Cells distributed in each stage were quantified as a percentage (*n* = 5 per group). *p < 0.05, **p < 0.01, and ***p < 0.001 vs. CTRL (control, untreated) group.

To examine the potential growth-inhibiting effect of maltol on melanoma, we investigated the influence of maltol on the cell proliferation of B16F10 cells. Maltol treatment decreased the propagation of B16F10 cells in a concentration-dependent manner (*p* < 0.05; Figure 2A). Notably, stronger inhibition was observed with maltol concentrations of 50 μg/mL and above. To investigate any synergistic effects between maltol and the anti-cancer drug cisplatin, cisplatin was simultaneously treated with maltol at concentrations of 50, 100, and 150 μg/ml. As expected, the results from the MTT assay revealed that co-treatment with maltol and cisplatin exhibited a more potent inhibitory effect on viability compared with the individual maltol or cisplatin treatments (*p* < 0.05; Figure 2B). Maltol caused cell cycle arrest in G2/M phase in the flow cytometric analysis of cell cycle distribution (*p* < 0.05; Figures 2C, D). In addition, the co-treatment displayed a more effective induction of G2/M phase cell cycle arrest compared to either treatment in isolation (*p* < 0.05; Figures 2C, D). Furthermore, we verified that maltol significantly suppressed the viability in A375 melanoma cells, and that the combination with cisplatin also had a synergistic effect on viability (Supplementary Figure S1).

### Pro-apoptotic effect of maltol in B16F10 melanoma cells

**FIGURE 3:**
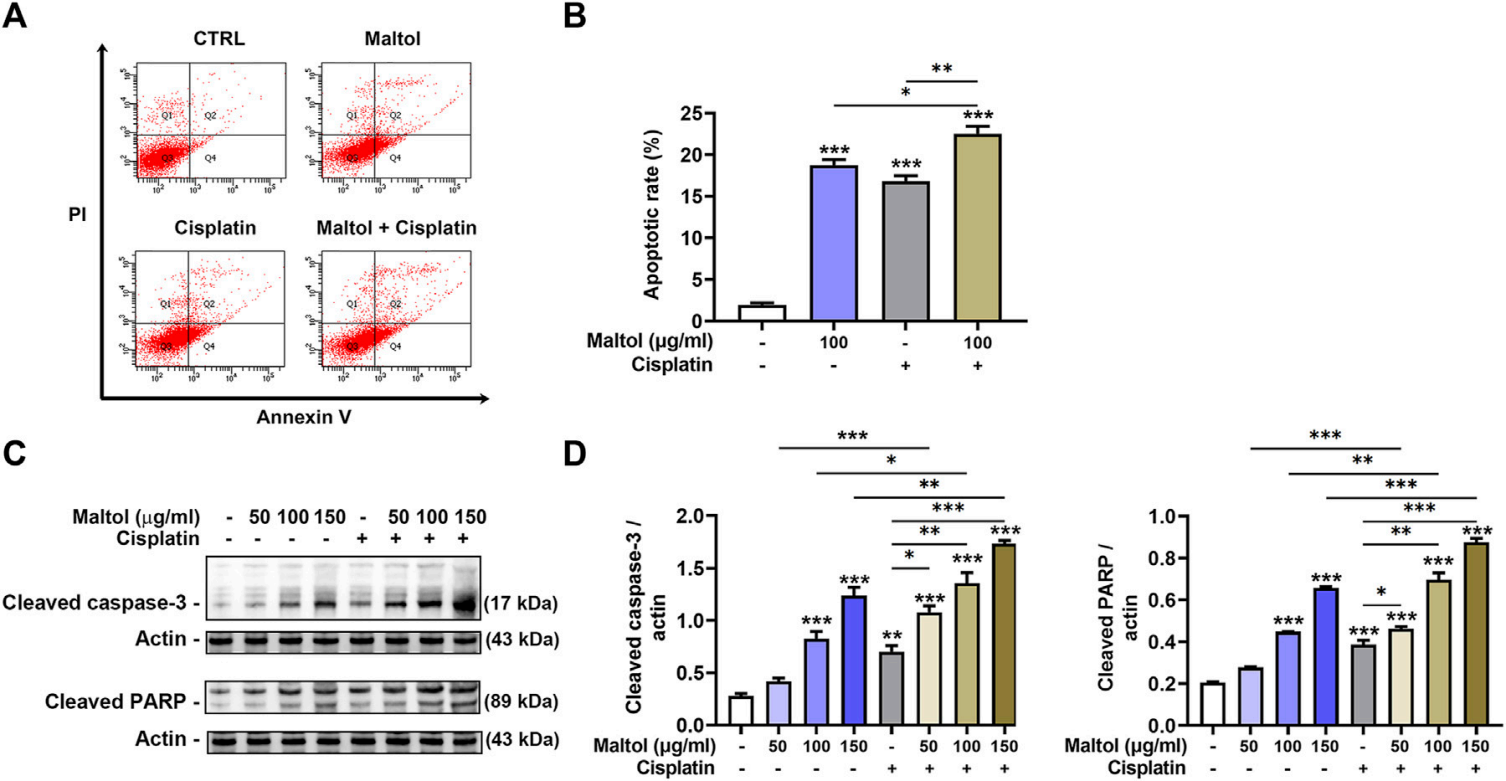
Effects of maltol on apoptosis of B16F10 melanoma cells. **(A)** Maltol or cisplatin (20 µM) were treated in B16F10 cells for 48 h. The cells were stained with annexin V and PI before flow cytometric analysis. **(B)** Apoptosis was quantified as a percentage (*n* = 4 per group). **(C)** Maltol or cisplatin were treated in B16F10 cells for 24 h. The cleaved caspase-3 and cleaved PARP levels were assessed by Western blot analysis. Actin was a loading control. **(D)** Graphs show quantification of relative band intensities for cleaved caspase-3 and cleaved PARP (*n* = 3 per group). *p < 0.05, **p < 0.01, and ***p < 0.001 vs. CTRL (control, untreated) group.

We then performed flow cytometric analysis to assess the amount of apoptotic cells in maltol-treated melanoma cells using annexin V and PI double staining. As shown in Figures 3A, B, maltol significantly increased in the percentages of apoptotic rates (*p* < 0.05). Correspondingly, the co-treatment with maltol and cisplatin led to a significant enhancement when compared either treatment alone (*p* < 0.05). Furthermore, Western blotting analysis indicated that maltol significantly increased the cleaved caspase-3 and PARP levels compared to the control samples, indicating pro-apoptotic activities (*p* < 0.05; Figures 3C, D). Similarly, the combined treatment showed a significant synergistic effect on these levels (*p* < 0.05; Figures 3C, D).

### Regulatory effect of maltol on IFN-γ-induced PD-L1 expression in B16F10 melanoma cells

**FIGURE 4:**
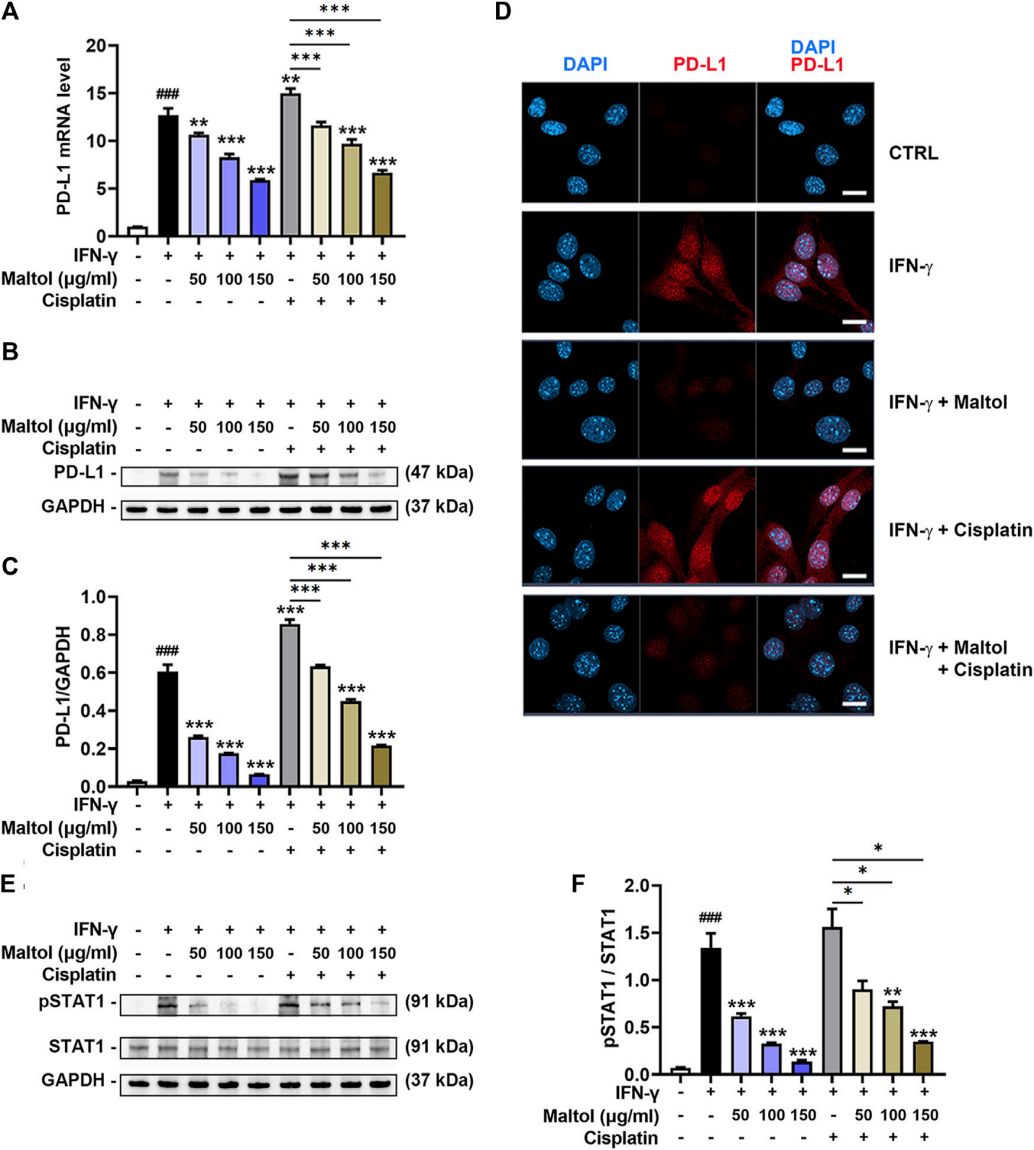
Effects of maltol on IFN-γ-induced PD-L1 expression in B16F10 melanoma cells. **(A)** Maltol or cisplatin (20 µM) was pre-treated, and IFN-γ (10 ng/mL) was then treated for 24 h in B16F10 cells. The mRNA expression was detected by real-time qPCR analysis (*n* = 5 per group). Each mRNA was normalized to GAPDH mRNA. **(B)** The PD-L1 level was analyzed by Western blot analysis. GAPDH was a loading control. **(C)** Graphs show quantification of relative band intensities for PD-L1 (*n* = 3 per group). **(D)** Representative micrographs indicate PD-L1 staining in immunofluorescence analysis (scale bar = 20 μm). **(E)** Maltol or cisplatin was pre-treated and IFN-γ was then treated for 10 min in B16F10 cells. The pSTAT1 level was analyzed by Western blot analysis. GAPDH was a loading control. **(F)** Graphs show quantification of relative band intensities for pSTAT1 (*n* = 3 per group). ^###^ *p* < 0.001 CTRL (control, untreated) group. *p < 0.05, **p < 0.01, and ***p < 0.001 vs. IFN-γ treated group.

Since the influence of maltol on the PD-L1 expression in tumor cells has not yet been determined, we examined the impact of maltol on the IFN-γ-induced upregulation of PD-L1 expression in B16F10 cells. As shown in Figure 4A, results from PCR experiments indicated that IFN-γ stimulated the mRNA levels of PD-L1, while maltol substantially attenuated the increase in PD-L1 (*p* < 0.05). Several studies have found that cisplatin upregulates PD-L1 expression, inducing resistance to cisplatin-induced apoptosis in tumor models (Tran et al., 2017; Hu et al., 2021). In line with previous reports, cisplatin significantly enhanced the PD-L1 mRNA expression upregulated by IFN-γ, and maltol apparently suppressed this increase (*p* < 0.05; Figure 4A). Furthermore, we detected a significant inhibition of the protein expression levels of PD-L1 from maltol-treated B16F10 cell lysates compared to the control group and the cisplatin-treated group, respectively, using Western blotting analysis (*p* < 0.05; Figures 4B, C). We next conducted immunofluorescence assays to confirm the impact of maltol on PD-L1 expression upregulated by IFN-γ. Similar to the results from the immunoblotting analysis, maltol remarkably reduced IFN-γ-induced or both IFN-γ and cisplatin-induced fluorescence signal enhancements in PD-L1 staining (Figure 4D). We next conducted flow cytometric analysis to investigate PD-L1 levels on the melanoma cell surface. Maltol significantly attenuated IFN-γ-induced membrane PD-L1 expression (*p <* 0.05; Supplementary Figure S2). The increase in PD-L1 expression by IFN-γ is transcriptionally regulated through activation of STAT1 in tumor cells (Xu et al., 2018). As expected, results from the immunoblotting analysis showed that the STAT1 phosphorylation levels increased by IFN-γ or both IFN-γ and cisplatin were significantly inhibited by maltol (*p <* 0.05; Figures 4E, F). Additionally, we verified that maltol significantly suppressed the increase in PD-L1 mRNA expression by IFN-γ or both IFN-γ and cisplatin in A375 melanoma cells (Supplementary Figure S3).

### Regulatory effect of maltol on T cell-regulated melanoma cell killing in CTLL-2 cells

**FIGURE 5:**
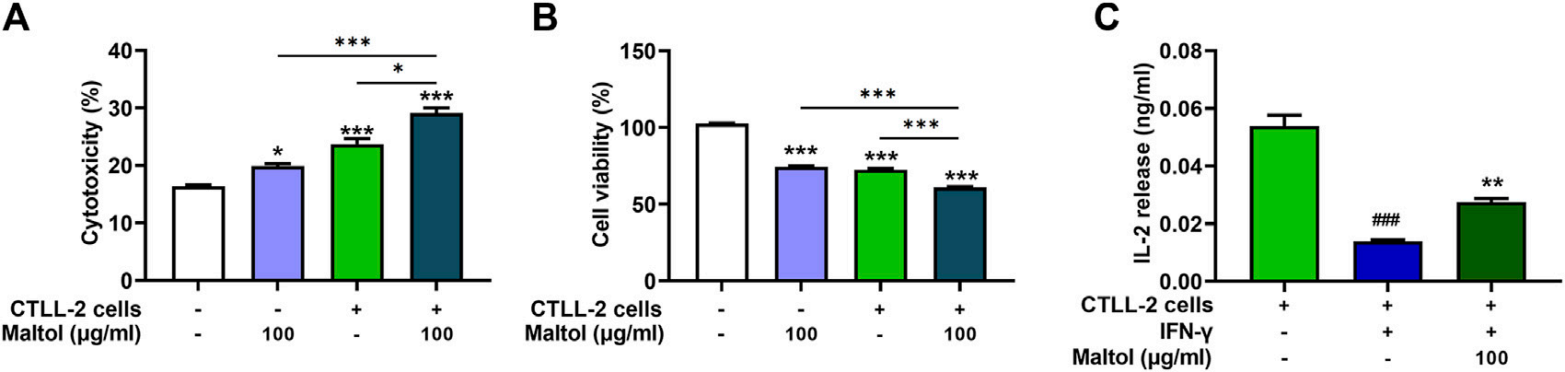
Effects of maltol on CTLL-2-mediated B16F10 killing. B16F10 cells were co-cultured with CTLL-2 cells with effector-to-target ratios of 20:1. **(A)** The cytotoxicity of CTLL-2 cells to B16F10 cells was measured with an LDH detection assay kit (*n* = 3 per group) and **(B)** viability of remaining B16F10 cells was assessed using an MTT assay (*n* = 4 per group). *p < 0.05, **p < 0.01, and ***p < 0.001 vs. CTRL (control, untreated) group. **(C)** IL-2 level secreted from co-cultured cells was measured by ELISA (*n* = 4 per group). ^###^ *p* < 0.001 CTRL (control, untreated) group. ***p < 0.001 vs. IFN-γ treated group.

CTLs are the most powerful effectors in anti-tumor immune responses and have the capacity to directly kill tumor cells (Raskov et al., 2021). We assessed the influence of maltol on T cell-regulated melanoma cell killing, specifically using PD-1-expressing CTLL-2 cells (Chen et al., 2021). To evaluate the modulatory effect of maltol on CTLL-2 cell-regulated melanoma cell killing, we measured the cytotoxicity exerted by CTLL-2 cells and subsequent cell viability after co-culture with B16F10 cells. Maltol significantly increased the total activity of lactate dehydrogenase (LDH) induced by CTLL-2 cells, suggesting severe damage to B16F10 cells (*p <* 0.05; Figure 5A). Correspondingly, the results from the cell viability assay showed that the decrease in cell viability caused by CTLL-2 cells was strongly enhanced by maltol (*p <* 0.05; Figure 5B). IL-2 is important for CTLs activity (Seo et al., 1993; Hidalgo et al., 2005). Thus, we further analyzed a regulatory effect of maltol on IL-2 release from the co-cultured cells. The IL-2 level was reduced in the IFN-γ-treated co-cultured cells, whereas maltol increased the diminished IL-2 levels (*p* < 0.05; Figure 5C).

## Discussion

**FIGURE 6:**
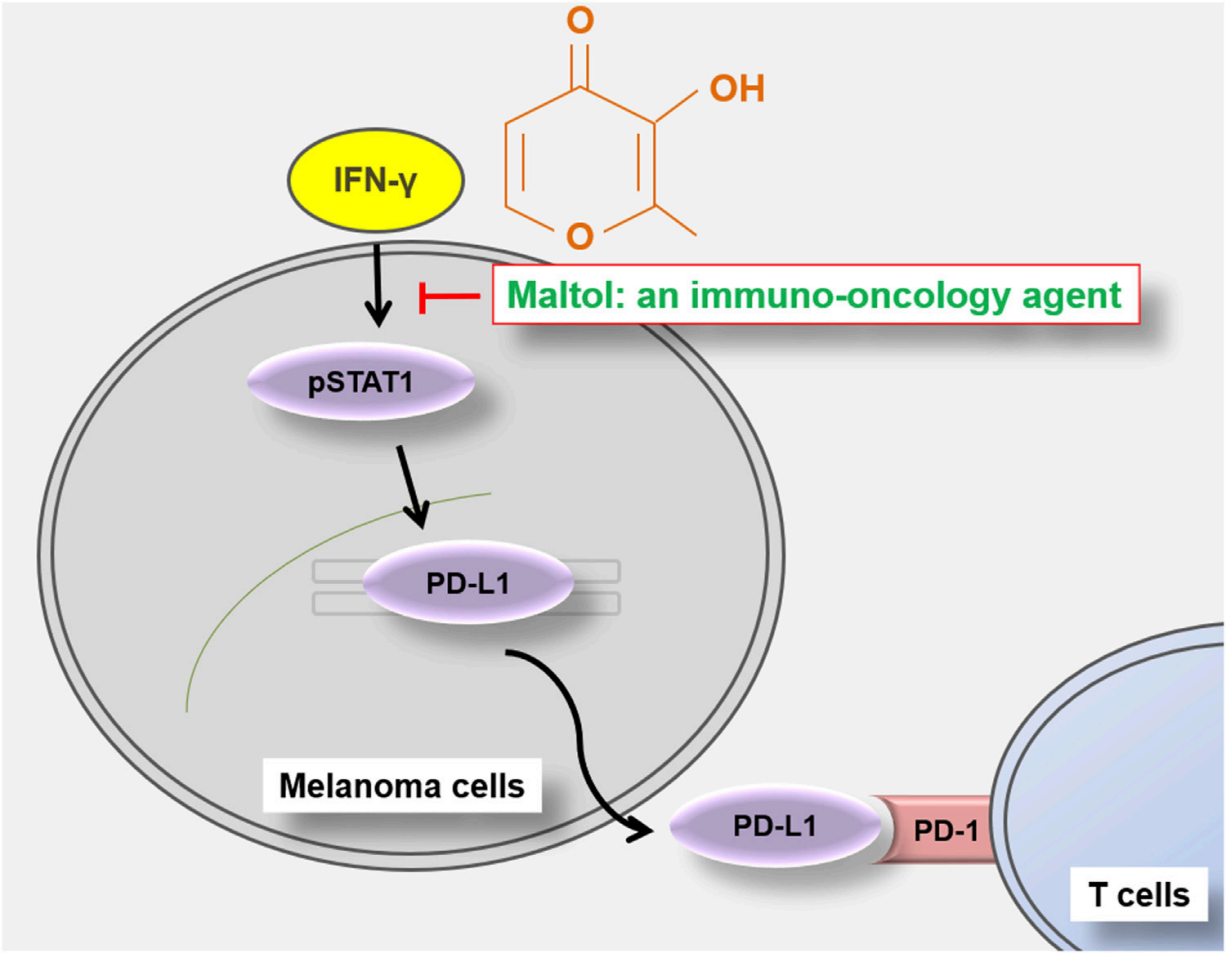
Schematic representation of maltol-mediated anti-melanoma effects in B16F10 melanoma cells.

In the present study, we identified that maltol has anti-cancer effects via modulating the PD-L1 signaling pathway in melanoma. Maltol arrested the B16F10 cell cycle with a pro-apoptotic effect. Maltol inhibited IFN-γ-induced PD-L1 expression through the downregulation of STAT1 phosphorylation. In addition, maltol enhanced T cell-regulated melanoma cell eradication with an increase in IL-2 production (Figure 6).

Melanoma is the most aggressive skin cancer and originates in melanocytes. The B16 melanoma model is the most widely used metastatic melanoma model in preclinical studies (Voltarelli et al., 2017). B16F10 cells have been established to analyze the potential of immuno-oncology agents and to support the development of novel therapeutics (Nakamura et al., 2002). Growing evidence has shown the potential anti-cancer effects against melanoma by utilizing various natural products in B16F10 cells (Gao et al., 2016; Yu et al., 2020). The current work showed that maltol exhibits anti-cancer effects through suppressing the cell viability of B16F10 cells. This result may support previous reports demonstrating the inhibition of B16F10 cells progression by *Panax ginseng* extract containing maltol (Yun et al., 2015). Thus, we suggest a promising effect of maltol on melanoma that may have potential clinical implications.

The amount of cleaved caspase-3 observed in the Western blot did not align with the results from annexin V staining; however, in the present study, maltol promoted apoptotic rates with an increase in levels of cleaved caspase-3. Apoptosis is associated with multiple proteins, including PARP, Bax, Bad, p21, p53, cytochrome c, and AIF (Sim et al., 2020). The pro-apoptotic influence of maltol may involve not only caspase-3 but also a range of apoptosis-related proteins. More studies are needed to further demonstrate the entire apoptotic mechanism associated with maltol.

Immunotherapy based on PD-1/PD-L1 pathway blockade represents a groundbreaking therapeutic approach and has been demonstrated to be effective in the treatment of several cancers (Teng et al., 2018). Clinical studies have shown that the therapeutic efficacy of blockade of the PD1-PD-L1 checkpoint is related to PD-L1 expression in tumor cells (Wang et al., 2016; Zhang et al., 2018; Wang et al., 2021). High PD-L1 levels may also serve as a selective marker to stratify patients suitable for PD1-PD-L1 checkpoint blockade therapy (Yi et al., 2023). In this work, we found the suppressive effect of maltol on both mRNA and protein expression levels of PD-L1 after IFN-γ stimulation in B16F10 cells. Moreover, this work provides insight into underlying mechanisms governing maltol’s effect on PD-L1 expression by showing that maltol inhibits STAT1 phosphorylation. Thus, we suggest that maltol has an anti-cancer effect by targeting PD-L1 in melanoma.

Intriguingly, our immunofluorescence experiments showed that PD-L1 was expressed in the nucleus. PD-L1, a plasma membrane multifunctional protein, can be translocated to the nucleus through interaction with multiple proteins. The nuclear presence of PD-L1 in several melanoma cell lines, including B16F10, could result from exogenous DNA damage, phosphorylation, acetylation, or other post-translational modifications (Gao et al., 2020; Kornepati et al., 2022; Lee et al., 2022). Nuclear PD-L1 accelerates tumorigenesis, confers resistance towards anti-PD1/PD-L1 therapies, and regulates cancer immune evasion and immunotherapy in the tumor microenvironment (Gao et al., 2020; Xiong et al., 2021; Yu et al., 2023). Consequently, the change in cellular localization of PD-L1 might be caused by interactions of multiple proteins in the *in vitro* environment of this work. However, in the future, the change in cellular localization of PD-L1 in melanoma needs to be explored more.

Cisplatin chemotherapy has formed the standard systemic therapy for various cancers (Tran et al., 2017; Grabosch et al., 2019). However, several studies have reported an increase in tumor PD-L1 expression caused by cisplatin, leading to resistance against cisplatin-induced apoptosis and subsequent immune evasion (Tran et al., 2017; Grabosch et al., 2019; Hu et al., 2021). Thus, those authors have proposed that the combination of cisplatin with anti-PD-L1 antibodies or substances down-regulating PD-L1, might enhance cisplatin’s anti-cancer response (Tran et al., 2017; Grabosch et al., 2019; Hu et al., 2021). In accordance with previous evidence, we showed that the combined application of maltol and cisplatin reversed cisplatin-induced PD-L1-mediated chemotherapy and immunotherapy resistance, resulting in synergistic effects in terms of growth inhibition and pro-apoptotic action. Consequently, we propose that maltol can be applied as a combinatorial agent to overcome PD-L1-related chemotherapy resistance, including in the context of cisplatin treatment.

To verify the viability of maltol as an anti-cancer agent, cell cycle arrest and apoptosis were simultaneously analyzed at equivalent concentrations. Although significant, the degree of the combination effect with cisplatin was inconsistent between the two assays. Several studies have demonstrated variable anti-cancer efficacy at the same concentration across these assays (Ghosh et al., 2018; Luo et al., 2019; Hu et al., 2020). We therefore propose the application of maltol against PD-L1-mediated resistance to cisplatin. However, a comprehensive array of growth and apoptosis-related studies on the combination is needed for better understanding.

CTLs efficiently eliminate cancer cells (Weigelin et al., 2021). High PD-L1 levels prevent CTLs from targeting tumor cells (Yi et al., 2023). The reduction of PD-L1 in melanoma cells contributes to the efficacy of CTLs-mediated cancer cell elimination (Jung et al., 2022). CTLL-2 cells, which are mouse-derived CTLs, are often used as a CTL model (Kametaka et al., 2003). An immuno-oncology agent was proposed by analyzing the effector cells (CTLL-2)-mediated cytotoxicity against target cells (B16F10) simultaneously with PD-L1 expression reduction (Jung et al., 2022). PD-L1/PD-1 checkpoint-mediated immune suppression is accompanied by reduced IL-2 production, and blockade of the PD-L1/PD-1 interaction restores T cell functionality along with increased IL-2 levels (Xu et al., 2018). IFN-γ signaling can impair CTL function by reducing IL-2 production (Tau et al., 2001; Hidalgo et al., 2005). In this work, maltol potentiated CTLL-2 cells-regulated B16F10 killing and IL-2 production. Thus, maltol could hold promise as an immuno-oncology agent, acting by inhibiting the expression of PD-L1 while simultaneously enhancing the function of CTLs.

## Conclusion

In conclusion, our findings reveal, for the first time, that maltol has an anti-melanoma effect by liberating PD-L1/PD-1 checkpoint-regulated immunosuppression, leading to more efficient T cell killing, and being effective against PD-L1-mediated immunotherapy resistance associated with cisplatin. Therefore, maltol, as an immuno-oncology agent, could be used as a potential candidate for combating melanoma. However, future studies are needed to investigate whether maltol’s impact extends not only CTLs but also to PD-L1-expressing and tumor-related antigen-presenting cells.

## Data Availability

The original contributions presented in the study are included in the article/Supplementary Material, further inquiries can be directed to the corresponding author.
