# Supplementary material for: Maltol has anti-cancer effects via modulating PD-L1 signaling pathway in B16F10 cells
Source: Front Pharmacol. 2023 Sep 5;14:1255586. doi: 10.3389/fphar.2023.1255586 (PMC10508342; doi:10.3389/fphar.2023.1255586)
Supplement: Supplementary file 1 [file DataSheet1.docx]

**Maltol has anti-cancer effects via modulating PD-L1 signaling pathway in B16F10 cells**

**Na-Ra Han^1,2^, Hi-Joon Park^3^, Seong-Gyu Ko^2,4^ and Phil-Dong Moon^5*^**

^1^College of Korean Medicine, Kyung Hee University, Seoul, Republic of Korea, ^2^Korean Medicine-Based Drug Repositioning Cancer Research Center, College of Korean Medicine, Kyung Hee University, Seoul, Republic of Korea, ^3^Department of Anatomy & Information Sciences, College of Korean Medicine, Kyung Hee University, Seoul, Republic of Korea, ^4^Department of Preventive Medicine, College of Korean Medicine, Kyung Hee University, Seoul, Republic of Korea, ^5^Center for Converging Humanities, Kyung Hee University, Seoul, Republic of Korea

**SUPPLYMANTRY DATA**

**FIGURES**

**Figure S1** The cell viability of A375 cells

**Figure S2** The detection of PD-L1 in B16F10 cell membrane

**Figure S3** **Effects of maltol on IFN-γ-induced PD-L1 expression in A375 cells**


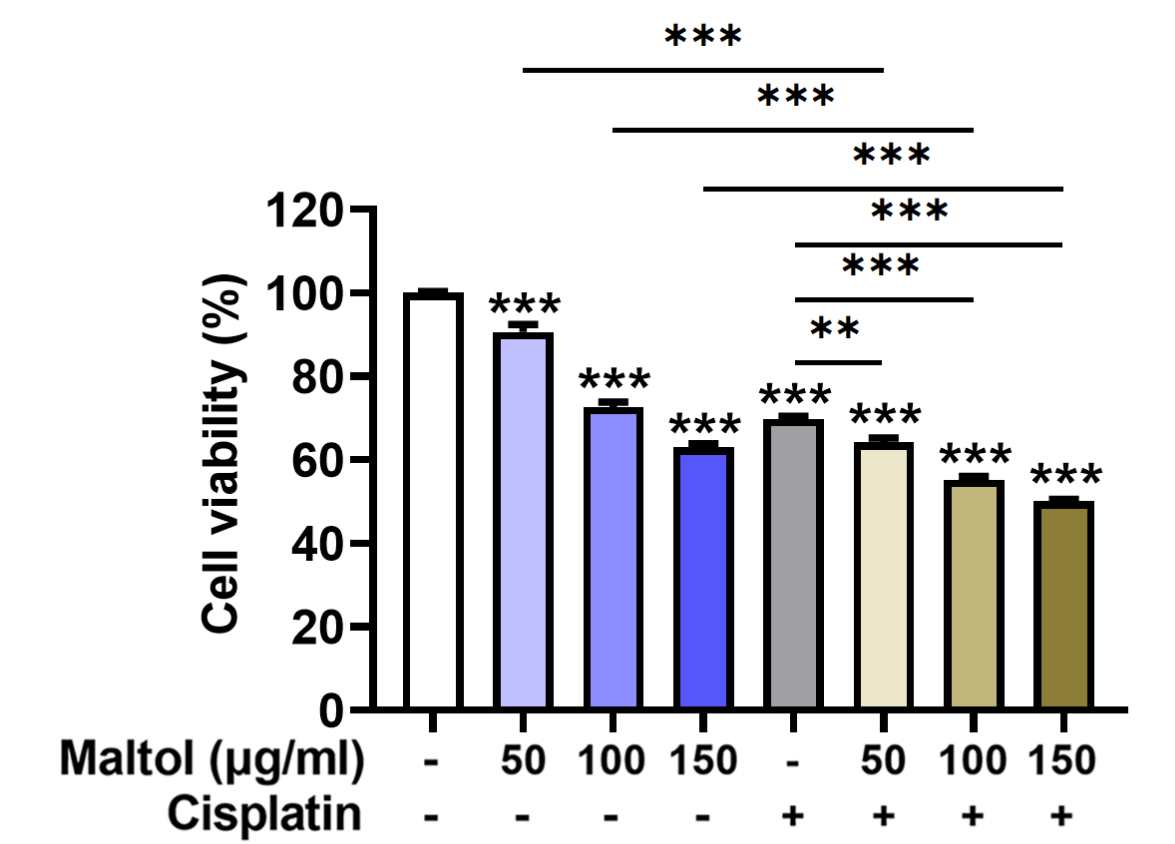


**Fig S1** The cell viability of A375 cells. Maltol and cisplatin (20 µM) were treated in A375 cells for 48 h. The cell viability was measured with an MTT assay (n = 6 per group). ^**^***p* < 0.01 and ^***^*p* < 0.001 vs CTRL (control, untreated) group.**


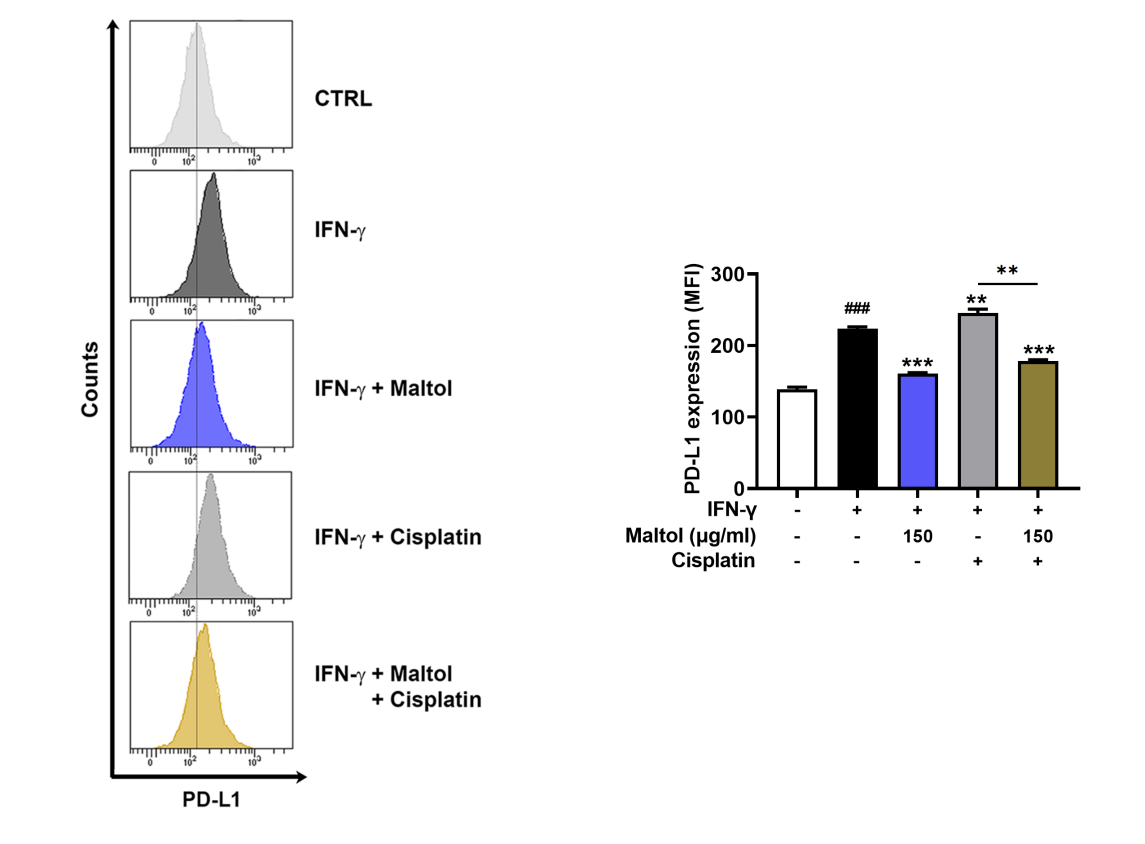


**Fig S2** The detection of PD-L1 in B16F10 cell membrane. Maltol or cisplatin (20 µM) was pre-treated and **IFN-γ** (10 ng/ml) was then treated for 24 h in **B16F10 cells**. The cell membrane surface PD-L1 expression was assessed by flow cytometry (n = 4 per group). MFI, median fluorescence intensity. ^###^*p* **< 0.001 CTRL (control, untreated) group.** ^**^***p* < 0.01 and ^***^*p* < 0.001 vs IFN-γ treated group.**


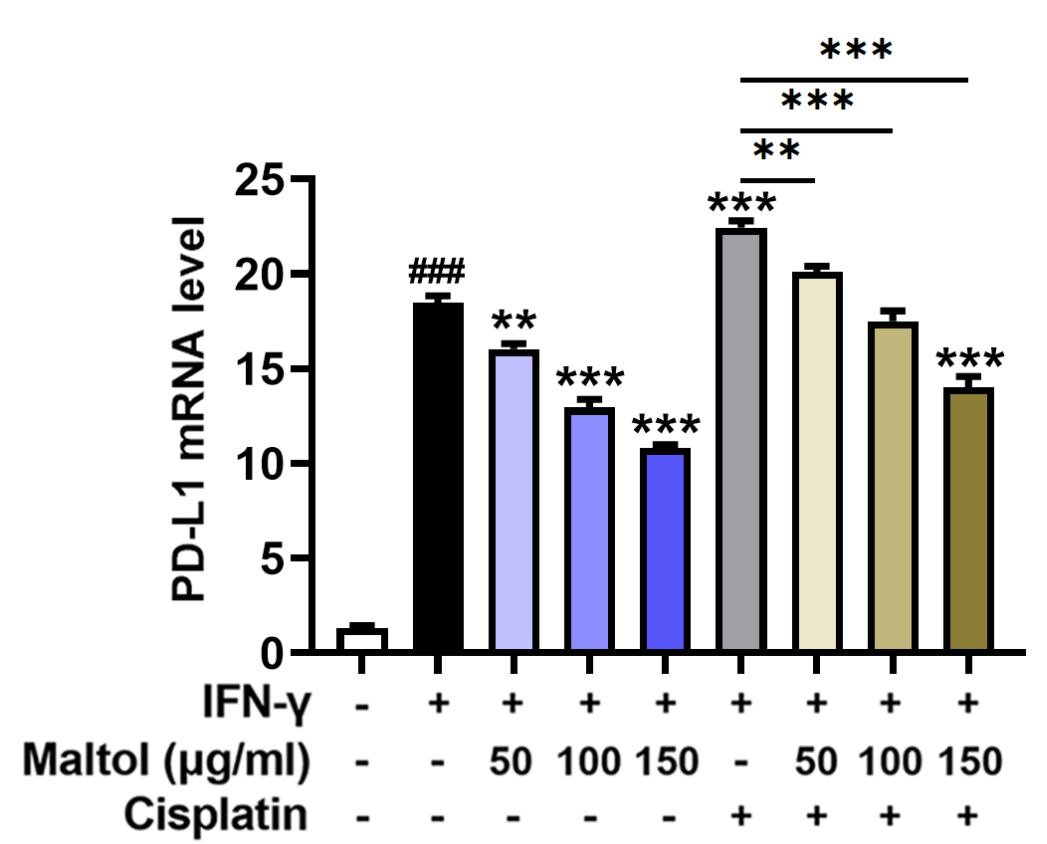


**Fig S3 Effects of maltol on IFN-γ-induced PD-L1 mRNA expression in A375 cells**. Maltol or cisplatin (20 µM) was pre-treated and **IFN-γ** (10 ng/ml) was then treated for 24 h in **A375 cells**. The mRNA expression was detected by real-time qPCR analysis (n = 5 per group). ^###^*p* **< 0.001 CTRL (control, untreated) group.** ^**^***p* < 0.01 and ^***^*p* < 0.001 vs IFN-γ treated group.**
